# Broadening the Differential of Pseudo–Atrioventricular Block

**DOI:** 10.1016/j.jaccas.2026.108126

**Published:** 2026-05-20

**Authors:** Federica Vernacchia, Luigi Sciarra, Sabina Gallina, Giuseppe Bagliani, Fabrizio Ricci

**Affiliations:** a Department of Neuroscience, Imaging and Clinical Sciences, “G. d’Annunzio” University of Chieti-Pescara, Chieti, Italy; b Department of Life, Health and Environmental Sciences, University of L'Aquila, L'Aquila, Italy; c Arrhythmology Unit, Cardiology Department, Foligno General Hospital, Foligno, Perugia, Italy; d University Cardiology Division, Heart Department, SS. Annunziata Polyclinic, Chieti, Italy; e Institute for Advanced Biomedical Technologies, “G. d’Annunzio” University of Chieti-Pescara, Chieti, Italy

**Keywords:** artifacts, conduction disorders, ECG, pseudo-AV block, signal saturation

## Abstract

**Case Summary:**

A 65-year-old man with no known cardiovascular disease and no prior history of syncope presented to the emergency department with fatigue, abdominal pain, and fever and was admitted with a diagnosis of sepsis of abdominal origin. On initial evaluation, he was hemodynamically stable and denied cardiac symptoms. A resting 12-lead electrocardiogram (ECG) showed conduction abnormalities of uncertain significance, prompting a focused interpretative challenge.

**Take-Home Messages:**

Transient ECG signal saturation can create pseudo–conduction abnormalities that closely mimic advanced atrioventricular block. Evaluation of internal signal coherence is essential to differentiate true electrophysiologic disorders from acquisition-related artifacts.

## Graphical Abstract

**Figure:**
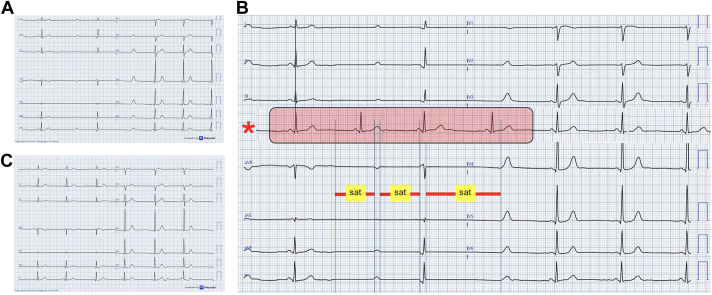

## Question

**Figure 1:**
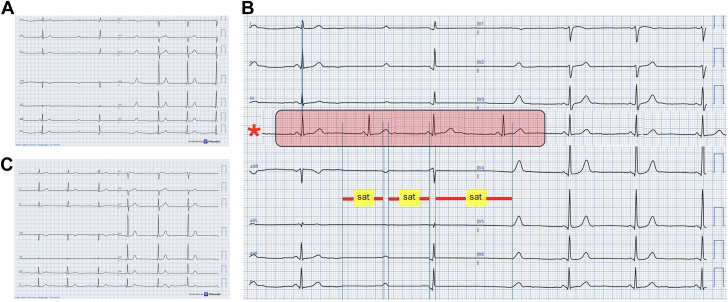
ECG Findings and Reconstruction (A) Admission 12-lead electrocardiogram (ECG). (B) Magnified view of the ECG shown in (A). The red shaded region indicates a manually reconstructed segment of the tracing, replacing a period of apparent electrical inactivity. Vertical markers labeled “sat” identify intervals of transient ECG signal saturation, during which ventricular depolarization was not recorded, resulting in a perfectly flat, noise-free isoelectric baseline preceding a normally contoured T wave. The red asterisk marks the reconstructed lead, in which the missing electrical activity during saturation has been reintegrated to restore a physiologically coherent depolarization–repolarization sequence. The continuous lead II rhythm strip displayed at the bottom facilitates assessment of atrioventricular relationships. (C) Repeat 12-lead ECG demonstrating normal atrioventricular conduction without recurrence of the previously observed artifacts.

What is noted on the ECG (Figure 1A)?

A. Paroxysmal atrioventricular block due to heightened vagal tone
B. Second-degree atrioventricular block, Mobitz type II, with junctional escape rhythm
C. High-grade atrioventricular block requiring urgent pacing
D. Sinus bradycardia with concealed junctional premature beats
E. Pseudo–conduction abnormality from transient ECG signal saturation
Answer: E

**Take-Home Message**

• Pseudo–atrioventricular block from ECG signal saturation should be suspected when an apparent pause is perfectly flat, noise-free, and lacks internal signal coherence.
• ECG patterns that violate basic electrophysiologic principles should prompt scrutiny for nonphysiologic signal loss and confirmation of resolution after systematic troubleshooting, helping avoid unnecessary downstream monitoring or pacing.

## Discussion

The ECG demonstrated sinus bradycardia, with a mean heart rate of approximately 40 beats/min. On initial inspection, a second-degree atrioventricular block, Mobitz type II, with a junctional escape beat was suspected.

Closer inspection, however, revealed several internally inconsistent features. In all leads, the second cardiac cycle appeared abnormal, with an apparent P wave followed by a markedly prolonged PR interval and a QRS complex displaying an atypical morphology. Most striking was the presence of an unusually prolonged isoelectric ST segment, terminated by a normally shaped T wave. Notably, the isoelectric line during this interval was perfectly linear and geometrically flat—devoid of noise—in sharp contrast to the low-amplitude baseline oscillations seen elsewhere in the tracing. This finding raised immediate concern for an ECG artifact.

Applying classic electrocardiographic reasoning, attention was drawn to what appeared to be an apparently isolated T wave, best appreciated in the precordial leads and in the DII lead continuous rhythm tracing at the bottom. From an electrophysiologic standpoint, ventricular repolarization cannot occur in the absence of preceding depolarization. The presence of a completely flat, noise-free isoelectric segment preceding this wave further supported a technical rather than biological explanation.

Such morphology is consistent with transient signal loss due to saturation of the ECG acquisition system, in which the amplifier is temporarily driven outside its operational voltage range, resulting in failure to record electrical activity. Under this interpretation, the apparent nonconducted P wave and PR interval prolongation were reinterpreted as artifacts reflecting brief signal dropout rather than true atrioventricular conduction delay. Reconstruction of the tracing (Figure 1B) by reintegrating the missing electrical activity restores a physiologically coherent sequence and excludes an underlying conduction abnormality.

ECG signal saturation is a transient recording phenomenon in which the acquisition system exceeds its dynamic operating range and the amplifier temporarily fails to register incoming cardiac electrical signals. It most often arises from overload of the right-leg drive amplifier, abrupt changes in electrode impedance, inadequate grounding, cable instability, or increased skin–electrode resistance related to diaphoresis or movement. On the tracing, it appears as a sudden, perfectly linear, noise-free isoelectric segment that interrupts otherwise coherent electrical activity. Recognition relies on identifying this internally inconsistent pattern and confirming resolution after systematic troubleshooting, including electrode reassessment; optimization of skin contact, particularly at the right-leg electrode; verification of cable connections; and repeat ECG acquisition.

This case exemplifies how technical artifacts may convincingly mimic cardiac conduction disorders. ECG signal saturation, although uncommon, may occur due to transient electrical disturbances, grounding issues, or amplifier overload, leading to apparent electrical silence or wave distortion.^1^ When intermittent, such artifacts may closely resemble high-grade atrioventricular block or escape rhythms. Careful appraisal of the internal coherence of the ECG signal remains essential to discriminate true pathology from artifacts^2^, ^3^, ^4^ or concealed junctional ectopy.^5^ Only a rigorous, deductive approach—grounded in electrophysiologic principles and knowledge of ECG acquisition systems—allows recognition of such patterns as nonphysiologic. Notably, ECG patterns that violate basic electrophysiologic principles, such as repolarization without preceding depolarization, should prompt immediate scrutiny for nonphysiologic signal abnormalities.

Following treatment of the abdominal infection, the patient recovered and was discharged in good clinical condition. Serial ECGs and 24-hour Holter monitoring showed normal conduction without recurrence of the previously observed abnormalities (Figure 1C).

## Funding Support and Author Disclosures

The authors have reported that they have no relationships relevant to the contents of this paper to disclose.
